# In-hospital care, complications, and 4-month mortality following a hip or proximal femur fracture: the Spanish registry of osteoporotic femur fractures prospective cohort study

**DOI:** 10.1007/s11657-018-0515-8

**Published:** 2018-09-14

**Authors:** Daniel Prieto-Alhambra, Carlen Reyes, Miguel Sanz Sainz, Jesús González-Macías, Luis Gracia Delgado, Cristina Alonso Bouzón, Sarah Mills Gañan, Damián Mifsut Miedes, Eduardo Vaquero-Cervino, Manuel Francisco Bravo Bardaji, Laura Ezquerra Herrando, Fátima Brañas Baztán, Bartolomé Lladó Ferrer, Ivan Perez-Coto, Gaspar Adrados Bueno, Jesús Mora-Fernandez, Teresa Espallargas Doñate, Jorge Martínez-Iñiguez Blasco, Ignacio Aguado-Maestro, Pilar Sáez-López, Monica Salomó Doménech, Vicente Climent-Peris, Ángel Díez Rodríguez, Humberto Kessel Sardiñas, Óscar Tendero Gómez, Jordi Teixidor Serra, José Ramón Caeiro-Rey, Ignacio Andrés Cano, Mariano Barrés Carsi, Iñigo Etxebarria-Foronda, Juan Dionisio Avilés Hernández, Juan Rodriguez Solis, Oscar Torregrosa Suau, Xavier Nogués, Antonio Herrera, Adolfo Díez-Perez

**Affiliations:** 1 GREMPAL (Grup de Recerca en Epidemiologia de les Malalties Prevalents de l’Aparell Locomotor) Research Group, CIBERFES, IDIAP Jordi Gol (Universitat Autònoma de Barcelona) and Instituto de Salud Carlos III, Av Gran Via de les Corts Catalanes, 587, Atic, 08007 Barcelona, Spain; 2 Musculoskeletal Pharmaco and Device Epidemiology – Centre for Statistics in Medicine, Nuffield Department of Orthopaedics, Rheumatology, and Musculoskeletal Sciences, University of Oxford, Windmill Road, Oxford, OX3 7LD UK; 3 Musculoskeletal Research Unit, IMIM-Parc Salut Mar, CIBERFES, Universitat Autònoma de Barcelona, Doctor Aiguader 88, 08003 Barcelona, Spain; 4 IIS Aragón (Instituto de Investigación Sanitaria de Aragón), Hospital Universitario Miguel Servet, Padre Arrupe, s/n, 50009 Zaragoza, Spain; 5 IDIVAL (Instituto de Investigación Marqués de Valdecilla), HUMV (Hospital Universitario Marqués de Valdecilla), UC (Universidad de Cantabria), Av de Valdecilla sn, 39011 Santander, Cantabria Spain; 6 Hospital Universitario Reina Sofía de Cordoba, Av Menendez Pidal, 14004 Córdoba, Spain; 7 Geriatric Unit, Hospital Universitario de Getafe, Carr. De Madrid – Toledo, Km 12,500, 28905 Getafe, Madrid Spain; 8 Traumatology and Orthopaedics Unit, Hospital Universitario La Paz, Paseo de la Castellana, 261, 28046 Madrid, Spain; 9 Hospital Clínico de Valencia, Av de Blasco Ibáñez, 17, 46010 Valencia, Spain; 10 Complejo Hospitalario de Pontevedra, Av Montecelo, 0, 36164 Casas Novas, Pontevedra Spain; 11 Hospital Regional Universitario de Malaga, Av. de Carlos Haya, s/n, 29010 Málaga, Spain; 12 F.E.A of the Traumatology and Orthopaedics Unit, Hospital Clínico Universitario Lozano Blesa, Av. San Juan Bosco, 15, 50009 Zaragoza, Spain; 13 Geriatric Unit, Hospital Universitario Infanta Leonor, Gran Vía del Este, 80, 28031 Madrid, Spain; 14 Hospital Son Llàtzer, Carretera de Manacor, PQ 4 (Son Ferriol), 07198 Palma de Mallorca, Spain; 15 Hospital Universitario San Agustín, Camino de Heros, 6, 33401 Avilés, Asturias Spain; 16 Internal Medicine Unit, Hospital Infanta Cristina, Av. de Elvas, s/n, 06080 Badajoz, Spain; 17 Geriatric Unit, Hospital Clínico San Carlos, calle Prof. Martín Lagos s/n, 28040 Madrid, Spain; 18 Hospital Obispo Polanco, Av. Ruiz Jarabo, s/n, 44002 Teruel, Spain; 19 Hospital San Pedro, Calle Piqueras, 98, 26006 Logroño, La Rioja Spain; 20 Hospital Universitario Rio Hortega, Calle Dulzaina, 2, 47012 Valladolid, Spain; 21 Hospital Universitario Fundación Jiménez Díaz, IdiPAZ (Instituto de Investigación del Hospital La Paz), Madrid, Spain; 22 Corporación sanitaria Universitaria Parc Tauli, Parc Taulí, 1, 08208 Sabadell, Barcelona Spain; 23 Traumatology and Orthopaedics Unit, Hospital Públic Lluis Alcanyis de Xàtiva, Carretera Xátiva-Silla, Km 2, 46800 Xàtiva, Valencia Spain; 24 Traumatology and Orthopaedics Unit, Hospital Virgen del Puerto, Paraje Valcorchero, 10600 Plasencia, Cáceres Spain; 25 Geriatric Care Unit, Complejo Hospitalario Torrecárdenas, Calle Hermandad de Donantes de Sangre, 04009 Almería, Spain; 26 Hospital Universitari Son Espases, Carr. de Valldemossa, 79, 07120 Palma, Islas Baleares Spain; 27 Hospital Universitari Vall de Hebron, Passeig de la Vall d’Hebron, 119-129, 08035 Barcelona, Spain; 28 Traumatology and Orthopaedics Unit, Complejo Hospitalario Universitario de Santiago de Compostela, Rúa da Choupana, s/n, 15706 Santiago de Compostela, A Coruña Spain; 29 Hospital Puerta del Mar, Av. Ana de Viya, 21, 11009 Cádiz, Spain; 30 Hospital Universitari i Politècnic La Fe, Av de Fernando Abril Martorell, 106, 46026 València, Spain; 31 Department of Orthopaedic, Alto Deba Hospital, Arrasate-Mondragon, Gipuzkoa Spain; 32 Orthogeriatric Unit, Hospital Clínico Universitario Virgen de Arrixaca, Ctra. Madrid-Cartagena, s/n, 30120 El Palmar, Murcia Spain; 33 Geriatric Unit, Hospital Universitario de Guadalajara, Calle Donante de Sangre, s/n, 19002 Guadalajara, Spain; 34 Bone Metabolism Unit, Internal Medicine Unit, Hospital General Universitari d’Elx, Carrer Almazara, 11, 03203 Elche, Alicante Spain; 35 Internal Medicine Department IMIM (Hospital del Mar Medical Research), CIBER FES ISCIII, Universitat Autónoma de Barcelona, Barcelona, Spain; 36 Department of Surgery, Aragón Health Research Institute, University of Zaragoza, Zaragoza, Spain

**Keywords:** Hip fracture, Registries, Osteoporosis and patient care management

## Abstract

**Summary:**

We have characterised 997 hip fracture patients from a representative 45 Spanish hospitals, and followed them up prospectively for up to 4 months. Despite suboptimal surgical delays (average 59.1 hours), in-hospital mortality was lower than in Northern European cohorts. The secondary fracture prevention gap is unacceptably high at 85%.

**Purpose:**

To characterise inpatient care, complications, and 4-month mortality following a hip or proximal femur fracture in Spain.

**Methods:**

Design: prospective cohort study. Consecutive sample of patients ≥ 50 years old admitted in a representative 45 hospitals for a hip or proximal femur fragility fracture, from June 2014 to June 2016 and followed up for 4 months post-fracture. Patient characteristics, site of fracture, in-patient care (including secondary fracture prevention) and complications, and 4-month mortality are described.

**Results:**

A total of 997 subjects (765 women) of mean (standard deviation) age 83.6 (8.4) years were included. Previous history of fracture/s (36.9%) and falls (43%) were common, and 10-year FRAX-estimated major and hip fracture risks were 15.2% (9.0%) and 8.5% (7.6%) respectively. Inter-trochanteric (44.6%) and displaced intra-capsular (28.0%) were the most common fracture sites, and fixation with short intramedullary nail (38.6%) with spinal anaesthesia (75.5%) the most common procedures. Surgery and rehabilitation were initiated within a mean 59.1 (56.7) and 61.9 (55.1) hours respectively, and average length of stay was 11.5 (9.3) days. Antithrombotic and antibiotic prophylaxis were given to 99.8% and 98.2% respectively, whilst only 12.4% received secondary fracture prevention at discharge. Common complications included delirium (36.1 %) and kidney failure (14.1%), with in-hospital and 4-month mortality of 2.1% and 11% respectively.

**Conclusions:**

Despite suboptimal surgical delay, post-hip fracture mortality is low in Spanish hospitals. The secondary fracture prevention gap is unacceptably high at > 85%, in spite of virtually universal anti-thrombotic and antibiotic prophylaxis.

**Electronic supplementary material:**

The online version of this article (10.1007/s11657-018-0515-8) contains supplementary material, which is available to authorized users.

## Introduction

Due to an increase in life expectancy [1], the burden of hip fractures is expected to reach 319 million fractures worldwide by 2040 [2], which poses a social and economic challenge for health care providers. Hip fractures are associated with an increased mortality and disability; mortality increases up to 33% at the end of the first year [3] and disability has been estimated at 5964 DALYs per 1,000 individuals [4]. In Europe, osteoporotic fractures account for a higher loss of years due to disability than most cancers [5]. Moreover, the stress of having a hip fracture affects not only the patient (due to the pain, the need of surgery and the usual long in-hospital stays) but also their family members and caregivers [6].

In this context, improving hip fracture care is becoming increasingly important for health care providers. Fracture patients are often frail and present many previous comorbid conditions [7]; hence, their management frequently requires long hospital stays and a complex process [8, 9]. For this reason, many hospitals have shifted towards a multi-disciplinary team to take care of these patients, which includes several specialities such as orthopaedists, general medicine, anaesthesiologists, rehabilitation, geriatricians, social workers, and primary care practitioners [8].

Geographical variations of hip fracture, with Spain accounting with one of the lowest hip fracture rates in Europe [10], renders comparison difficult with other national hip fracture registries reports previously published. Moreover, there is a lack of prospective accurate information on the current hospital care received by hip fracture patients, as well as on the post-operative complications and overall survival in Spain [11]. We therefore aimed to characterise patients, inpatient care (including surgery, rehabilitation, and prophylaxis of complications and/or secondary fracture prevention), inpatient complications risk, and up to 4-month mortality in a prospective cohort of Spanish hip or proximal femur fracture patients.

## Methods

### Study design and setting

We conducted a multi-centric, prospective cohort study in a representative 45 hospitals from 15 autonomic regions from Spain.

### Participants

#### Inclusion criteria of contributing hospitals

Hospitals were eligible to participate in the study if they had an ortho-geriatric specialist or a medical doctor responsible for the coordination of inpatient care for hip fracture patients during their index hospital admission. The hospitals were selected considering not only their geographic representativeness but also the type/volume/size of hospital to maximise the representativeness of the study sample.

#### Inclusion/exclusion criteria of cases

All adults of at least 50 years old, presenting with a fragility fracture of the hip or proximal femur at any of the participating hospitals during the recruitment period (June 2014 to June 2016) were invited up to a maximum of 30 consecutive patients per hospital. Participants (or their carers if they were unable to do so) signed consent and were included at the moment of hospital admission. From then on, they received usual care according to local protocols/practice.

A fragility fracture was defined as that produced by a low energy impact or without previous traumatism. Subjects with fractures due to neoplastic disorders (including primary or metastatic bone cancer) or in an irradiated site, traffic accidents, produced by falls from a height higher than 1.80 m, peri-prosthetic or distal femur fractures, and those with a previous ipsilateral femur fracture were excluded. Additionally, patients who for any medical or psychological reason were unable to receive usual care, or those who were already participating in related (with anti-osteoporosis drug/s or fracture care/surgery as intervention/s under study) clinical trial/s were also ineligible.

### Follow-up

All subjects were followed up for up to 4 months after their index admission date.

### Study outcomes

The main measurements of this study were socio-demographic features of the patient (age, sex, body mass index, civil status, ethnic and educational background, place of residence previous to fracture), comorbid conditions such as previous fractures, diagnose of osteopenia, osteoporosis, rheumatoid arthritis, and secondary osteoporosis (which was confirmed if the patient had any of the following disorders related to osteoporosis: type 1 diabetes, adult osteogenesis imperfecta, hyperthyroidism or premature menopause (< 45 years old), chronic malnutrition or malabsorption, or chronic liver disease). Falls (number of falls in the last year), date of menopause, medications (anti-osteoporosis medication), fracture risk factors (FRAX tool), characteristics of the fracture episode (circumstances, site, and type of fracture), in-patient care received (pre-operative assessment, type of anaesthesia, ASA (American Society of Anaesthesiologists) physical status classification system) grade, surgery, the post-operative management (early mobilisation, constipation prevention or need or urinary catheterisation) and rehabilitation, prophylaxis of post-surgery complications, and secondary fracture prevention measures, type of multi-disciplinary units involved (group of health care professionals including physiotherapy, occupational therapy, nursery and medicine) who are responsible of the assessment and treatment of hip fracture patients) as well as baseline and post-fracture clinical outcomes (inpatient complications and up to 4-month mortality) were also collected.

### Statistical methods

A descriptive analysis was conducted for all measurements; continuous variables were summarised as mean, median, standard deviation, and quartiles (inferior, superior, minimum, maximum). The number and percentage of patients in each group was reported for each categorical variable. Kaplan-Meier curves were computed to depict post-fracture mortality up to 4 months of follow-up and after stratification per age and sex. All the analyses were stratified by sex, age, previous fractures history, and type of fracture.

## Results

### Baseline characteristics of study participants and contributing hospitals

A total of 997 subjects admitted to 45 hospitals were included between June 2014 and June 2016, of whom 856 (85.9%) completed 4 months of follow-up, 99 (9.9%) died in the same period, and 42 (4. 2%) were lost to follow up. Baseline characteristics of the study population are reported in Table 1. In brief, participants were mostly widowed Caucasian old women, with primary education levels, who lived in the community until they fractured. Key fracture risk factors were common, including previous fractures (36.5%) and at least one fall in the previous year (43%). Ten-year absolute fracture risk assessed using the FRAX tool was estimated at a mean (standard deviation) of 15.2% (9.0%) and 8.5% (7.6%) for major osteoporotic and hip fracture respectively. Regarding the characteristics of contributing hospitals, median (inter-quartile range) volume was of 290 (200–370) hip fractures per annum, and dedicated staff included a median (inter-quartile range) of 12 (7–15) clinicians.

**Table 1:** Baseline characteristics of the population and fractures

Baseline characteristics of the population included		
Variable		*N* (%)
Age at fracture, mean (SD)		83.59 (8.4)
BMI, mean (SD)		25.17 (4.3)
Sex (women)		765 (76.7)
Current smoker		48 (4.9)
Alcohol drinking > 3 units/day		35 (3.6)
Ethnic background (Caucasian)		993 (99.6)
Education (level achieved)	None	199 (20.1)
	Basic/primary	673 (67.9)
	Secondary	89 (9.0)
	University degree	30 (3.0)
	Missing data	6
Civil status	Single	79 (8.0)
	Married	322 (40.6)
	Divorced	23 (2.3)
	Widow	564 (57.1)
	Missing data	9
Previous residence before fracture	Own home	817 (82.1)
	Hospital	2 (0.2)
	Care home	173 (17.4)
	Unknown	3 (0.3)
	Missing data	2
ASA grade	Healthy person	55 (5.6)
	Mild systemic disease	335 (33.8)
	Severe systemic disease	486 (49.0)
	Severe systemic disease that is a constant threat to life	115 (11.6)
	Missing data	6
Previous fracture	Hip	101 (10.2)
	Spine (clinical)	72 (7.3)
	Any fracture	364 (36.5)
Rheumatoid arthritis		20 (2.0)
Secondary osteoporosis		57 (5.8)
Parental hip fracture history		79 (8.1)
Steroid user		38 (3.9)
Falls	0	566 (56.8)
	1	134 (13.4)
	2	124 (12.4)
	3	71 (7.1)
	4 or more	100 (10.3)
FRAX	Major osteoporotic fracture risk, mean (SD)	15.17 (8.95)
	Hip fracture risk, mean (SD)	8.49 (7.63)
Baseline characteristics of the fractures		
Fracture type	Fragility	988 (99.1)
	Atypical femoral fracture	7 (0.7)
	Unknown	2 (0.2)
Circumstances at the time of fracture	Tripping	556 (56.1)
	Slipping	228 (23.0)
	Whilst sitting down	93 (9.4)
	Whilst lying in bed	28 (2.8)
	Other	198 (20.0)
Fracture site	Displaced, intra-capsular	279 (28.0)
	Non-displaced, intra-capsular	94 (9.4)
	Inter-trochanteric	444 (44.6)
	Sub-trochanteric	101 (10.1)
	Other	78 (7.8)

*BMI*, *Body Mass Index*; *ASA grade*, American Society of Anaesthesiologists physical status classification status; *Falls*, falls in the previous year; *FRAX*, fracture risk assessment tool

### Descriptive analysis

Fracture type and circumstances at the time of fracture are reported in Table 1. Hip fractures were predominantly either inter-trochanteric (44.6%) followed by intra-capsular displaced (28%), whilst 7 (0.7%) were classified as atypical femoral fractures. Most common circumstance/s leading to fracture were tripping (56.1%) or slipping (23%).

### In-patient care and management of the fracture

The in-patient pre-operative care and treatments as well as the characteristics of the surgery carried out are detailed in Table 2. Upon admission, 60.6% of the subjects were assessed using a multi-disciplinary protocol, and a medical doctor reviewed 61.9% of them previous to surgery. The three most common surgical procedures carried out were (in order) (1) internal fixation with a short intramedullary nail (38.6%), followed by (2) bipolar-cemented hemi-arthroplasty (15%), and (3_ internal fixation with long intramedullary nail (14.9%). In 75.5% of these surgeries, spinal anaesthesia was used, and almost all of the subjects received either antithrombotic or antibiotic prophylaxis (99.8% and 98.2% respectively).

**Table 2:** Inpatient pre-and post-operative care, characteristics of the surgery, and complications after the fracture

Pre-operative care/treatments		
Variable		*N* (%)
Multi-disciplinary assessment protocol upon admission		602 (60.6)
Medical/specialised nurse input	Geriatric assessment	352 (35.3)
	Assessed by a physician	617 (61.9)
	Specialised nurse	359 (37.4)
	Not assessed	109 (10.9)
Antibiotic prophylaxis (common regimen/s given)	Cephalosporines	886
	Other antibiotics	78
Anti-thrombotic prophylaxis	Pharmacological	981
	Mechanic/physical measures	164
Previous oral anticoagulants		124
Previous platelet inhibitor/s therapy		212
Surgery		
Anaesthesia (type of)	Only general	74 (7.5)
	General and nerve block	9 (0.9)
	General and spinal anaesthesia	15 (1.5)
	General and epidural anaesthesia	36 (3.7)
	Only spinal anaesthesia	743 (75.5)
	Spinal anaesthesia and nerve block	70 (7.1)
	others	37 (3.8)
	Missing data	13
Surgery (type of procedure)	Internal fixation-sliding hip screw	32 (3.2)
	Internal fixation-cannulated screw	33 (3.3)
	Internal fixation-long intramedullary nail	148 (14.9)
	Internal fixation-short intramedullary nail	384 (38.6)
	Unipolar hemiarthroplasty (non-cemented, non-coated)	35 (3.6)
	Unipolar hemiarthroplasty (non-cemented-coated with hydroxyapatite)	12 (1.2)
	Unipolar hemiarthroplasty (cemented)	76 (7.7)
	Bipolar hemiarthroplasty (non-cemented-non-coated)	8 (0.8)
	Bipolar hemiarthroplasty (non-cemented, coated with hydroxyapatite)	14 (1.4)
	Bipolar hemiarthroplasty (cemented)	149 (15.0)
	Arthroplasty-total hip replacement (non-cemented, non-coated)	11 (1.1)
Post-operative care/treatments		
Review by internal medicine/geriatrics (grade)	Consultant	666 (71.0)
	Senior resident doctor	61 (6.5)
	Junior resident doctor	13 (1.4)
	Not reviewed/assessed	186 (19.8)
	Head of department	12 (1.3)
	Missing data	59
Seen by falls prevention team		424 (42.7)
Multi-disciplinary team review/care		537 (54.1)
Early mobilisation		873 (87.8)
Prevention of constipation		657 (66.1)
Post-operative rehabilitation		592 (59.4)
Destination at discharge	Own home	490 (49.3)
	Care home	250 (25.1)
	Home hospitalisation	4 (0.4)
	Rehabilitation centre	138 (13.9)
	Long-term community hospital	78 (7.8)
	Others	17 (1.7)
Complications after the fracture		
Delirium		360 (36.1)
Urinary tract infection		97 (9.7)
Respiratory tract infection		80 (8.0)
Kidney failure		140 (14.1)
Heart failure		82 (8.2)
Pressure ulcers		36 (3.63)
Surgical wound infection		8 (0. 8)
Prosthesis or material infection		6 (0.6)
In-hospital mortality		21 (2.1)

Different multi-disciplinary models were observed in Spanish hospitals during the whole hospital stay of the patient. An 89.5% of the participating hospitals reported formally co-ordinated care with either internal medicine or geriatrics. A multi-disciplinary protocol was used to guide care in 76.3% of the cases, and the process was usually co-ordinated by specific staff members (either specialised nurse/s or medical doctors).

Regarding surgical delays and time to rehabilitation, these were on mean (standard deviation) of 59.1 (56.7) hours and 61.9 (55.1) hours respectively. Total length of stay was of mean (standard deviation) 11.5 (9.3) days.

Post-operative care/management and complications after the fracture are summarised in Table 2. The most common inpatient complications were delirium (36.1%) and kidney failure (14.1%). The total in-hospital mortality was 2.1%.

### Secondary fracture prevention

At discharge, bone health was reportedly not assessed for 23.6%, assessed but treatment deemed unnecessary/inappropriate for 20.5% of the participants. In addition, 14.9% were awaiting a bone health clinical assessment, and 3.2% were discharged pending a DXA scan before treatment. Regarding anti-osteoporosis medication/s, a 21.4% of the participants were prescribed anti-osteoporosis drug therapy at discharge (8.3% continuing previous therapy and the other 12.1% newly started treatments during hospital admission), and the percentage of such treatment increased but remained suboptimal at 32.1% and 37.8% at 1 and 4 months respectively.

### Mortality in the first 4 months after discharge

**Fig. 1:**
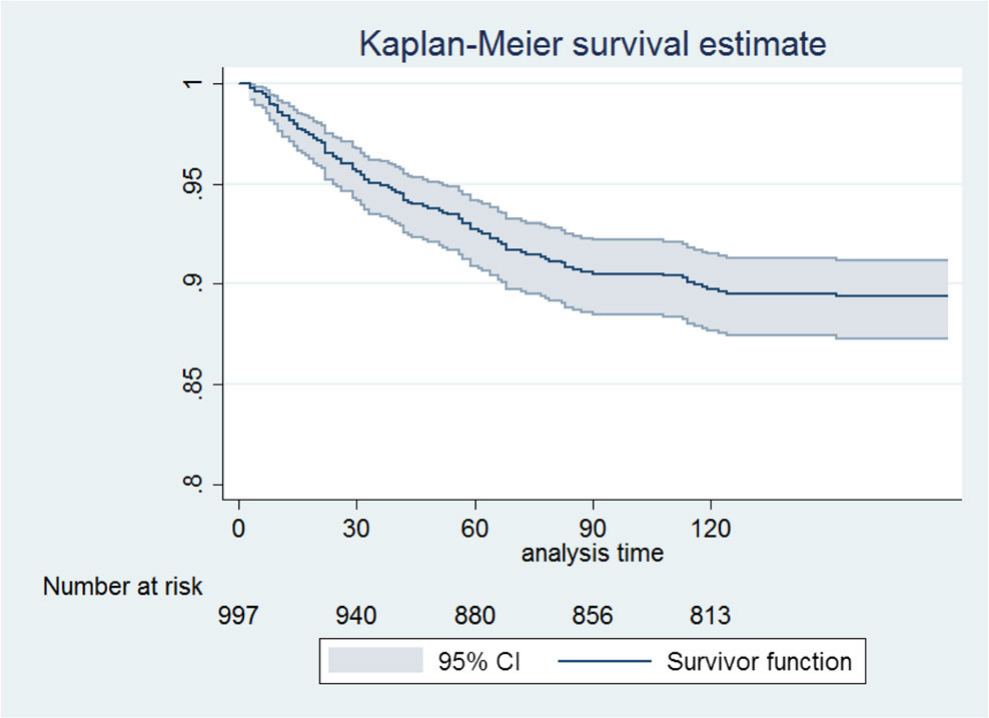
Kaplan-Meier estimates: cumulative mortality

**Fig. 2:**
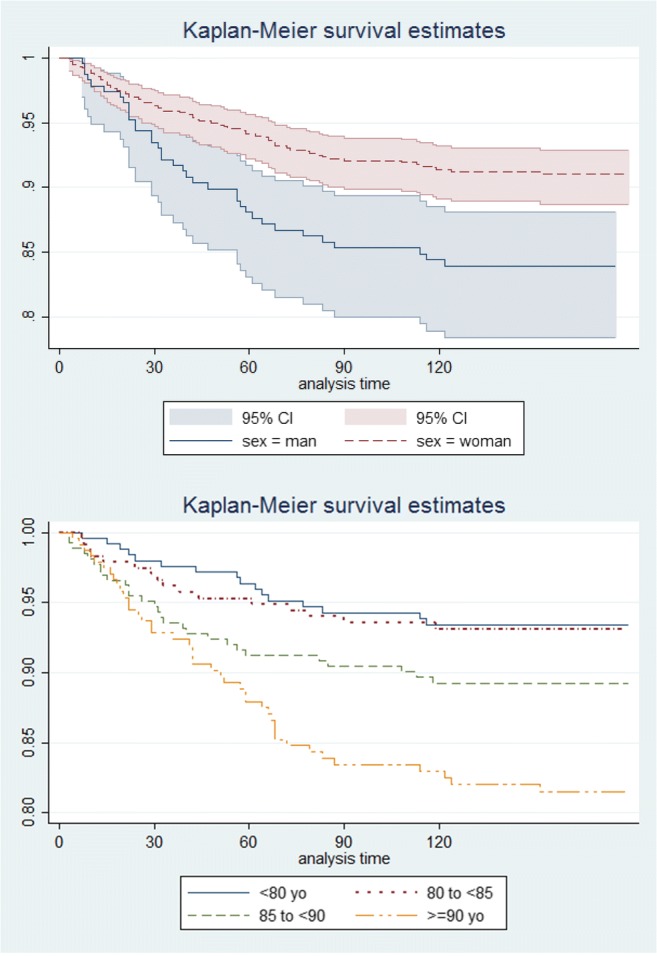
Kaplan-Meier estimates: cumulative mortality stratified by sex

Mortality in the first 4 months, overall, and stratified by sex is reported in Tables 3 and 4 and in Figs. 1 and 2. Kaplan-Meier survival estimates show an overall decreasing trend of mortality from the admission date to the end of the 4th month after the fracture, reaching 11% at the end of the 4th month. Results stratified by sex showed that men had an increased mortality after discharge compared to women (Table 4). When stratifying by age, we found that survival rates were similar in subjects up to 85 years old after which it drops drastically reaching 67% of survival rates among the oldest subjects (> 90 years old) at the end of this period (figure 3 in the supplementary material).

**Table 3:** Overall mortality up to 4 months of follow-up: life table

Interval (months)		*N* at end of interval	Deaths	Lost	Survival	95% conf. interval	
0	1	997	42	15	0.958	0.943	0.969
1	2	940	29	31	0.927	0.909	0.942
2	3	880	20	4	0.906	0.886	0.923
3	4	856	8	35	0.898	0.877	0.915

**Table 4:** Mortality up to 4 months of follow-up stratified by sex: life table

Interval (months)		*N* at end of interval	Deaths	Lost	Survival	95% conf. interval	
Men							
0	1	232	15	4	0.935	0.894	0.960
1	2	213	12	8	0.881	0.831	0.917
2	3	193	6	1	0.854	0.800	0.894
3	4	186	2	7	0.844	0.790	0.886
Women							
0	1	765	27	11	0.965	0.949	0.976
1	2	727	17	23	0.942	0.922	0.956
2	3	687	14	3	0.922	0.901	0.939
3	4	670	6	28	0.914	0.891	0.932

## Discussion

In this multi-centric, prospective, observational cohort study, we found that most of the subjects with hip fracture analysed were Caucasian elderly women with previous fracture/s, falls, and an intermediate to high 10-year fracture risk. Most of the fractures were inter-trochanteric and were produced by non-traumatic accidents. The majority of the subjects were first assessed through a multi-disciplinary protocol and followed afterwards either by a specific fall prevention team or by a multidisciplinary team.

Despite hospital delay in the surgery and rehabilitation, total length of stay was not affected (11.5 days).

The most common surgical procedure carried out was an internal fixation with short intra-medullary nail with spinal anaesthesia and the most frequent complications were delirium and kidney failure. Nearly all of the subjects were previously treated with either antithrombotic or antibiotic prophylaxis.

At discharge, the majority of the subjects returned to their own home and only one fifth of them were assessed for osteoporosis treatment. Hospital mortality remained low 2.1%, and there was an overall decreasing trend in the following 4 months after the fracture.

Hip fractures result in a socio-economic burden for health care systems and are expected to increase due to the ageing of the population. Therefore, optimising the treatment and care of these fractures is a top priority for health care providers [5]. Baseline characteristics of our population did not differ from the ones analysed in other recent hip fracture registries or audits [12–16]. As in our report, most of them were old women with an ASA score between 2 and 3; however, our results show that a large proportion of our subjects were at an increased risk of fracture at the time of admission; over 36% reported having a previous fracture and at least one fall in the last year.

The fracture risk assessment tool (FRAX) was also calculated and despite it has been validated in many countries including Spain, an underestimation of the fracture risk among Spanish women has been previously reported [17]. To overcome this, new thresholds have been proposed [3]; low risk < 5; intermediate risk ≥ 5 to < 7.5 and high risk ≥ 7.5. If we take these thresholds into account, our population would be considered a high-risk of fracture population; however, if we take into account the original thresholds of the FRAX (low risk < 10; intermediate risk ≥ 10 to < 20; high risk ≥ 20) our subjects would be in the intermediate risk for major osteoporotic fractures and low risk for hip fractures.

When analysing the characteristics of the fracture admitted to the hospital devices and the type of surgery carried out, the majority were inter-trochanteric fractures due to slipping or tripping, which differs from some of the studies published, especially in the USA and the UK [13–15], where the most frequent fractures were femoral neck fractures [14], non-intertrochanteric extra-capsular fractures [15], and intracapsular fractures [13]. Seven cases were classified as atypical hip fractures. Environmental differences, such as the weather or the pavement conditions, could lead to different fracture mechanisms and, therefore, to different type of fractures. The most common surgical procedure carried out in the hospitals analysed was an internal fixation with short intra-medullary nail, which is in agreement with the type of surgery used for these fractures in some of the other registries [14, 16].

Our study reported a delay in the time to surgery and in the time to the initiation of the rehabilitation (59 and nearly 62 h after the fracture admission respectively). The Catalan Government carried out another report in 2013 and the mean delay from admission to surgery in 68% of the hospitals analysed did not exceed the 48 h [7]. Possible explanations for this increase in the delay might be related to healthcare delivery and organisational factors. Median time to surgery was also reported in the UK registry [13] and was found to be lower than ours (24.5 h). Reasons for delay in our hospitals could be due to worse patient conditions previous to the surgery (e.g. comorbidities) or because of a lower number of material means and resources to carry on the surgeries (lower number of operating rooms or surgeons). However, a main limitation of the Patel et al. study [13] is that it is based on only 1 hospital (compared to the 48 hospitals were our study was carried out), limiting its external validity. Despite the delay in the surgery our in-hospital mortality rates (2.1%) were found to be lower than that reported in other countries [12–14]. International comparison of mortality rates is difficult, given that not all of them measured it at the same time.

Regarding the length of stay (LOS), there is a higher variability of results in the available literature [12–14, 16], leading to think that it could be due to overall health care system structure and fracture care practices in the various countries and the different population analysed. A recent meta-analysis and systematic review showed that multidisciplinary care models improved the patient outcomes in terms of LOS, in-patient and long-term mortality [8]. Our results are in accordance with the actual trends of implementing a multidisciplinary care; the majority of our hospitals carried out a multi-disciplinary protocol assessed by a physician and during the post-operative care, the assessment was carried out either by a specific fall prevention team or by a multidisciplinary team, depending on the hospital analysed.

Regarding the anti-osteoporosis medication, only 20.5% of the subjects in our population were assessed for osteoporosis treatment during their hospital stay and at discharge, only 12.4% received anti-osteoporosis medication, which increased up to 37.8% in the first 4 months after discharge. The low proportion of subjects with anti-osteoporosis medication has been previously reported [16]. In a report carried out by the Catalan Government in 2013 that aimed to analyse the healthcare procedures in this region [7] among subjects aged at least 65 years old who were hospitalised because of a femur fracture, patients who had a fracture consumed 10% less anti-osteoporotic medications than those without a fracture.

Despite that the anti-osteoporosis medication is recommended by the main Spanish Traumatology Society guidelines [9] especially among subjects at high risk of fracture such as those that have already sustained a hip fracture, less than 50% of our subjects were treated at the end of the 4th month after the hip fracture. This percentage might be higher if we add those that were already taking an osteoporosis treatment before the hip fracture.

This is an observational report and therefore is subject to the limitations of this type of study. However, other limitations need to be considered; first, we were unable to gather any information regarding the possible causes of delay of the surgery/rehabilitation or determine if the LOS was influenced by the pre-fracture comorbidities of the subjects. Second, by excluding subjects who could not follow the usual practice, we might have been introducing a selection bias; however, given that our population was not excluded neither because of their comorbidities nor because of their ability to answer the questionnaires, this selection bias is probably minimum. Moreover, we were also not able to draw any casual links between the surgery, type of pre-fracture or post-fracture care received, and their recovery in terms of LOS, complications, and functionality at discharge. Finally, the low mortality rates might be related with the criteria of participating centres’ selection, which had to have some kind of “medical care” for these patients. On the contrary, our study has several strengths, as are the prospective data collection, the wide representativeness of the participating centres, selected by size and geographical region, and the consecutive sampling of cases. All these elements contribute to the external validity of the data, minimising the likelihood of bias.

## Conclusions

Overall, the care of hip fractures admitted to Spanish hospitals seems in line with other registries published in recent years. The delays detected in the initiation of the surgery and rehabilitation did not affect the total length of stay or in the in-patient mortality, which were below what has been reported in other countries. As new models of care are created in order to give a better response to the patients’ needs during the care of the hip fracture, it is uncertain which model may work best. The multidisciplinary approach carried out by most of the Spanish hospitals seems to follow the latest trend in fracture patient care although secondary prevention treatment is still strikingly low.

## Electronic supplementary material

Fig. 3 (PNG 380 kb)

High resolution (TIF 784 kb)
