# Supplementary figures and images for: In-hospital care, complications, and 4-month mortality following a hip or proximal femur fracture: the Spanish registry of osteoporotic femur fractures prospective cohort study
Source: Arch Osteoporos. 2018 Sep 14;13(1):96. doi: 10.1007/s11657-018-0515-8 (PMC6153683; doi:10.1007/s11657-018-0515-8)

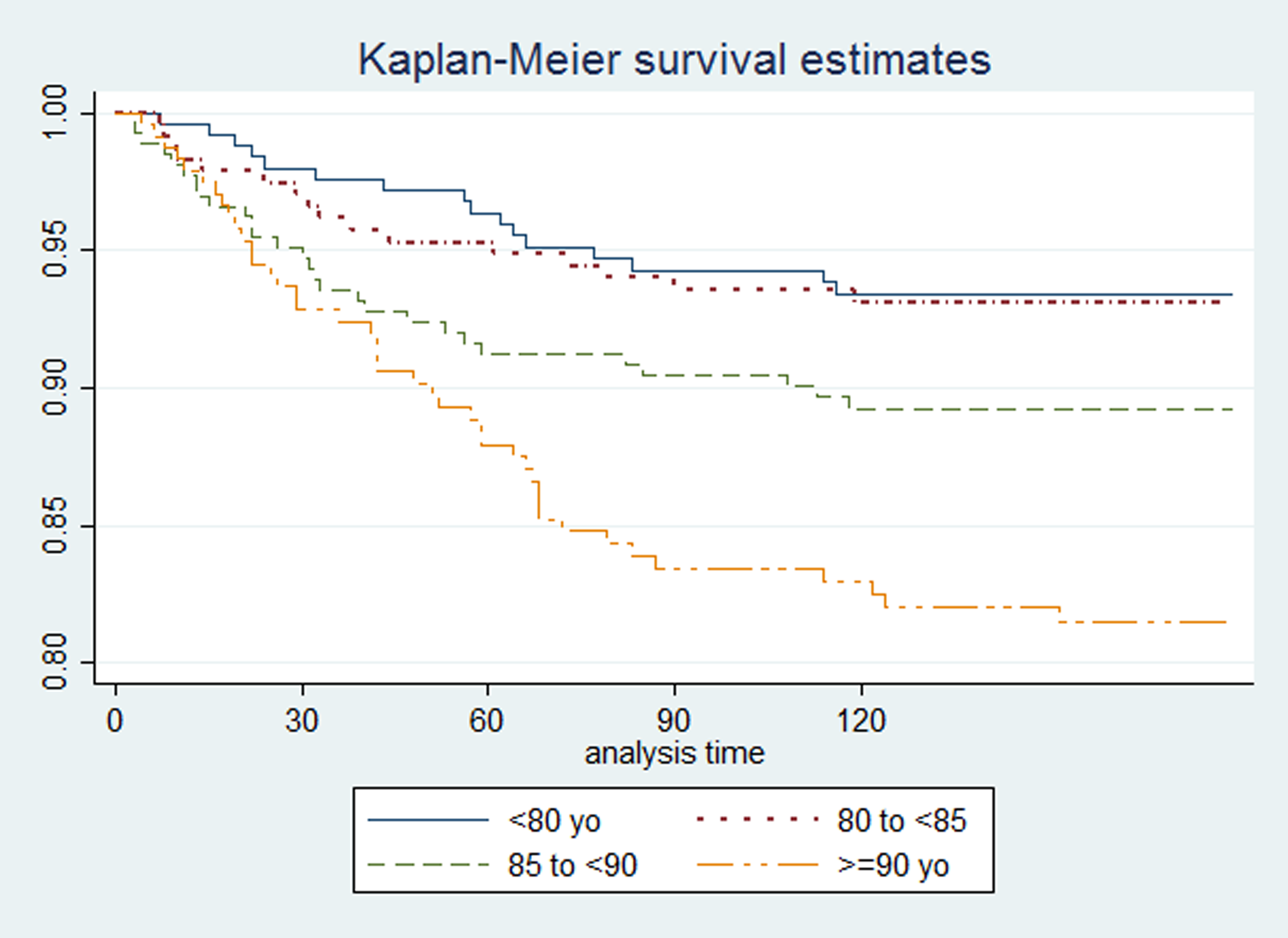

Supplement: Supplementary file 1 — (PNG 380 kb) [file 11657_2018_515_Fig3_ESM.png]

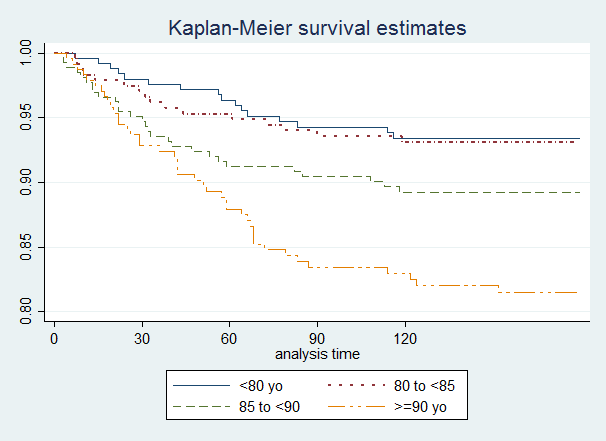

Supplement: Supplementary file 2 — High resolution (TIF 784 kb) [file 11657_2018_515_MOESM1_ESM.tif]
